# Characterisation of COPD heterogeneity in the ECLIPSE cohort

**DOI:** 10.1186/1465-9921-11-122

**Published:** 2010-09-10

**Authors:** Alvar Agusti, Peter MA Calverley, Bartolome Celli, Harvey O Coxson, Lisa D Edwards, David A Lomas, William MacNee, Bruce E Miller, Steve Rennard, Edwin K Silverman, Ruth Tal-Singer, Emiel Wouters, Julie C Yates, Jørgen Vestbo

**Affiliations:** 1 Thorax Institute, Hospital Clinic, IDIBAPS, Universitat de Barcelona; CIBER Enfermedades Respiratorias and Fundació Caubet-Cimera, Mallorca, Spain; 2 Department of Respiratory Medicine; University Hospital Aintree, Liverpool, UK; 3 Department of Respiratory Medicine; Brigham and Women's Hospital, Boston, MA, USA; 4 Department of Radiology, University of British Columbia, Vancouver General Hospital, Vancouver, BC, Canada; 5 GlaxoSmithKline, Research Triangle Park, NC, USA; 6 Department of Medicine, University of Cambridge, Cambridge Institute for Medical Research, Cambridge, UK; 7 University of Edinburgh & Royal Infirmary, Edinburgh, UK; 8 GlaxoSmithKline, King of Prussia, PA, USA; 9 University of Nebraska Medical Center, Omaha, NE, USA; 10 Department of Medicine; Brigham and Women's Hospital, Boston, MA, USA; 11 Department of Respiratory Medicine, Maastricht University Medical Centre, Maastricht, the Netherlands; 12 Department of Cardiology and Respiratory Medicine, Hvidovre Hospital/University of Copenhagen, Denmark, and University of Manchester, Manchester Academic Health Science Centre, UK

## Abstract

**Background:**

Chronic obstructive pulmonary disease (COPD) is a complex condition with pulmonary and extra-pulmonary manifestations. This study describes the heterogeneity of COPD in a large and well characterised and controlled COPD cohort (ECLIPSE).

**Methods:**

We studied 2164 clinically stable COPD patients, 337 smokers with normal lung function and 245 never smokers. In these individuals, we measured clinical parameters, nutritional status, spirometry, exercise tolerance, and amount of emphysema by computed tomography.

**Results:**

COPD patients were slightly older than controls and had more pack years of smoking than smokers with normal lung function. Co-morbidities were more prevalent in COPD patients than in controls, and occurred to the same extent irrespective of the GOLD stage. The severity of airflow limitation in COPD patients was poorly related to the degree of breathlessness, health status, presence of co-morbidity, exercise capacity and number of exacerbations reported in the year before the study. The distribution of these variables within each GOLD stage was wide. Even in subjects with severe airflow obstruction, a substantial proportion did not report symptoms, exacerbations or exercise limitation. The amount of emphysema increased with GOLD severity. The prevalence of bronchiectasis was low (4%) but also increased with GOLD stage. Some gender differences were also identified.

**Conclusions:**

The clinical manifestations of COPD are highly variable and the degree of airflow limitation does not capture the heterogeneity of the disease.

## Background

Chronic obstructive pulmonary disease (COPD) is defined by the presence of poorly reversible airflow limitation [1]. Yet, COPD is a complex, multi-component, heterogeneous disease, whose clinical, functional and radiological presentation varies greatly from patient to patient despite having a similar degree of airflow limitation [1-3]. Unfortunately, the prevalence, distribution and inter-relationships of the main clinical, functional and radiological manifestations of the disease in a large, well-characterised and controlled population of patients are lacking.

Evaluation of COPD Longitudinally to Identify Predictive Surrogate Endpoints (ECLIPSE) is a large observational study of COPD patients and controls conducted at 46 centres in 12 countries aimed at defining COPD phenotypes and identifying biomarkers and/or genetic parameters that help to predict disease progression [4]. ECLIPSE, therefore, offers a unique opportunity to characterise the heterogeneity of COPD. To this end, we present herein the cross-sectional analysis of the data collected at recruitment in ECLIPSE. Specifically, we sought: *(1) *to characterise the heterogeneity of COPD as a whole (vs. controls); *(2) *to explore the relationships (or lack of them) of the main clinical, functional and radiological characteristics of the disease; *(3) *to investigate the level of heterogeneity within each stage of disease severity, using either the classification proposed by Global initiative for chronic Obstructive Lung Disease (GOLD), which is based upon the degree of airflow limitation [1], or the BODE index, a multidimensional grading system that has proven better than the FEV_1 _at predicting the risk of death from any cause and from respiratory causes among COPD patients [5]; and, finally, *(4) *because the prevalence of COPD in women is increasing [6], we also analyzed potential gender differences in the clinical, functional and radiological variables studied.

## Methods

### Study design

The study design of ECLIPSE (Clinicaltrials.gov identifier NCT00292552; GSK study code SCO104960) has been published previously [4]. Briefly, ECLIPSE is an observational, longitudinal and controlled study where, after the baseline visit, subjects are evaluated at 3 months, 6 months and then every 6 months for 3 years. Results presented here represent the cross-sectional analysis of the data obtained at baseline. ECLIPSE complies with the Declaration of Helsinki and Good Clinical Practice Guidelines, and has been approved by the ethics committees of the participating centres. All participants provided written informed consent.

### Population

**Figure 1:**
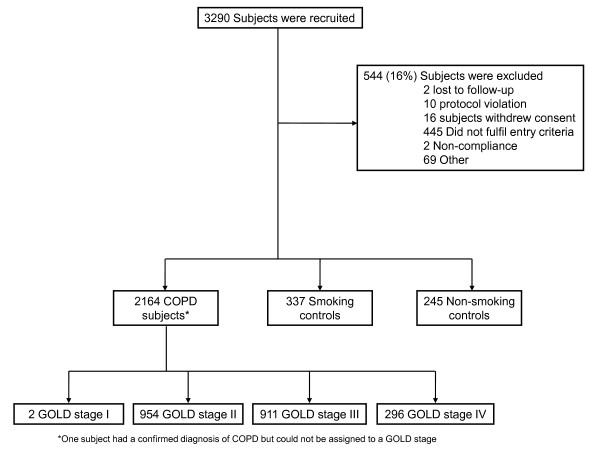
**Consort diagram**.

**Figure 2:**
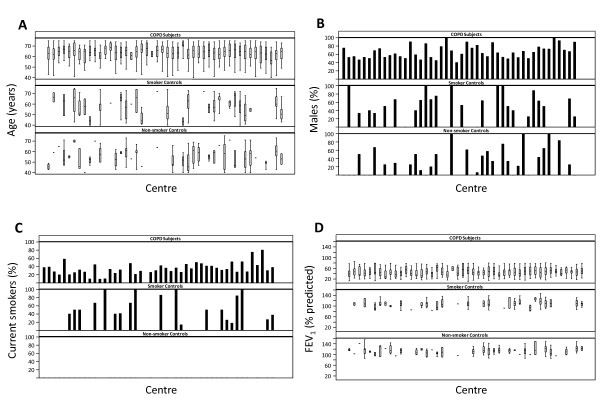
**Variability of age (panel A), gender (panel B), smoking status (panel C) and FEV_1 _(panel D) in the three groups of individuals recruited into ECLIPSE by the 46 participating centres**.

Power calculation was based on precision of effect estimates in COPD subgroups for rate of decline in FEV_1 _over 3 years (confidence interval width of at most 15 mL/year in rate of FEV_1 _decline). The sizes of the control groups were based on both the ability to detect a difference of at least 16.5 mL/year rate of decline in FEV_1 _between COPD patients and controls, and to detect a 50% increase in exposure (required 5-7 COPD patients per control) for any diagnostic test. Based upon these calculations, we studied 2164 patients with COPD (GOLD stage 2-4), 337 smoking controls and 245 non-smoking controls (Figure 1). Inclusion criteria were as follows [4]. ***COPD patients***: *(1) *Male/female subjects aged 40-75 years; *(2) *Baseline post-bronchodilator FEV_1 _< 80% of the reference value and FEV_1_/FVC ≤0.7; and, *(3) *Current or ex-smokers with a smoking history of ≥10 pack-years. ***Smoker controls***: (1) Male/female subjects aged 40-75 years, who are free from significant disease as determined by history, physical examination and screening investigations; *(2) *Baseline post-bronchodilator FEV_1 _> 85% of the reference value and FEV_1_/FVC > 0.7; and, *(3) *Current or ex-smokers with a smoking history ≥10 pack-years. ***Non smoking controls***: *(1) *Male/female subjects aged 40-75 years, who are free from significant disease as determined by history, physical examination and screening investigations; *(2) *Baseline post-bronchodilator FEV_1 _> 85% of the reference value and FEV_1_/FVC > 0.7; and, *(3) *Smoking history of <1 pack-year. Besides, all participants: *(4) *signed and dated their written informed consent prior to participation (which had been approved by the Ethics Committees of all participating institutions); and, *(5) *had to have the ability to comply with the requirements of the protocol and be available for study visits over 3 years. Key exclusion criteria were the presence of a respiratory disorder other than COPD, other significant inflammatory diseases or a reported COPD exacerbation within 4 weeks of enrolment [4]. COPD patients were recruited from the outpatient clinics of the participating centres (Figure 1). Smoker and non-smoker controls were recruited through site databases and other methods (advertisements in local newspapers and television/radio stations) where appropriate. Figure 2 presents the variability of age (panel A), gender (panel B), smoking status (panel C) and FEV_1 _(panel D) in the three groups of individuals recruited into ECLIPSE (COPD patients, smokers and non-smokers with normal lung function) by each of the 46 participating centres.

### Measurements

The American Thoracic Society (ATS) respiratory questionnaire, the modified Medical Research Questionnaire (mMRC) and the COPD-specific version of the St. George's Respiratory Questionnaire (SGRQ-C) [7] were used to record clinical data. Exacerbations requiring treatment with antibiotics, oral corticosteroids and/or hospitalisation in the year prior to the study were also recorded. Co-morbidities were self-reported using the ATS-DLD-78 questionnaire. Nutritional status was assessed by the body mass index (BMI) and fat-free mass index (FFMI), the latter measured by bioelectrical impedance [4]. Spirometry and the 6 minute walking distance (6MWD) were performed according to international guidelines [8,9]. Spirometric reference values were those of the European Community for Coal and Steel (ECCS) [10]. The BODE index was calculated according to Celli et al [5].

All subjects underwent a low-dose computed tomography (CT) scan of the chest acquired using multi-detector-row CT scanners (GE Healthcare or Siemens Healthcare) with a minimum of 4 rows, obtained in supine position at suspended full inspiration without administration of intravenous contrast. Exposure settings were 120 kVp and 40 mAs and images were reconstructed using 1.0 mm (Siemens) or 1.25 mm (GE) contiguous slices and a low spatial frequency reconstruction algorithm (GE: Standard; Siemens: b35f). CT scanners were calibrated regularly using industry and institutional standards. All of the CT scans were evaluated at the central imaging unit at the University of British Columbia, Vancouver, Canada. Quantitative assessment of lung volumes and the percentage of lung CT voxels below a threshold of -950 Hounsfield Units as a representative of the presence of emphysema, was performed using the software Pulmonary Workstation 2.0 (VIDA Diagnostics, Iowa City, IA, U.S.A.) [11]. Two radiologists also determined the presence or absence of bronchiectasis.

### Statistical analysis

Results are shown as mean ± SD, frequency distribution or proportion, as appropriate. Because none of the continuous variables were normally distributed (Kolmogorov-Smirnov test), Kruskal-Wallis tests were used to analyze the statistical significance of differences between groups. Differences in categorical variables were assessed using Cochran-Mantel-Haenszel tests. Correlations between variables of interest were explored using Spearman's Rho; p values less than 0.05 (two sided) were considered significant.

### Role of the funding source

The study was sponsored by GlaxoSmithKline. A Steering Committee and a Scientific Committee comprising in total ten academics and six representatives of the sponsor developed the original study design and concept, the plan for the current analyses, approved the statistical plan, had full access to the data, and was responsible for decisions with regard to publication. The study sponsor did not place any restrictions with regard to statements made in the final paper.

## Results

### COPD patients compared with controls

COPD patients were older than controls and had more pack years of smoking than smokers with normal lung function (Table 1). BMI was lower in patients with COPD but differences were negligible in absolute values, and the FFMI was not different between groups. Patients with COPD were more symptomatic (according to both the mMRC and SGRQ-C) than smokers with normal lung function or never smokers (Table 1). Co-morbidities were more prevalent in COPD; 38% of patients with COPD had more than one co-morbidity (23% in smokers with normal lung function and 16% in non-smokers; p < 0.001). By definition, patients with COPD had airflow limitation, whereas spirometry was normal in the two control groups. On average, patients with COPD showed more reversibility of airflow limitation after inhalation of a bronchodilator than controls (Table 1). The 6MWD in COPD was 369 ± 122 metres and the BODE index 3.2 ± 2.1 units. The amount of emphysema was significantly greater in COPD than in controls. Bronchiectasis was observed in 4% of patients with COPD but in none of the controls.

**Table 1:** Mean ± SD, median (IQR), or proportion of the main anthropometric, clinical, functional and radiological variables in the three groups of participants

	COPD (n = 2164)	Smoking controls (n = 337)	Non-smoking controls (n = 245)	p value
**Clinical data**				

Age (years)	63.4 ± 7.1^a, b^	55.4 ± 9.0	54.1 ± 9.0	< 0.001

Pack-years	48.6 ± 27.1 ^a, b^	31.6 ± 21.5^b^	0.2 ± 1.1	< 0.001

Current Smokers (%)	36^a^	61		< 0.001

BMI (kg/m^2^)	26.5 ± 5.7^b^	26.8 ± 4.6	27.7 ± 5.4	0.004

FFMI (kg/m^2^)	17.2 ± 2.8	17.1 ± 2.6	17.2 ± 2.7	0.842

mMRC Score	1.7 ± 1.1 ^a, b^	0.2 ± 0.5^b^	0.1 ± 0.3	< 0.001

SGRQ-C total score	50.1 ± 20.3 ^a, b^	9.6 ± 12.3^b^	4.8 ± 6.5	< 0.001

Number of exacerbations^c^	0.9 ± 1.2 ^a, b^	0.0 ± 0.1	0.0 ± 0.1	< 0.001

Heart trouble (%)	26 ^a, b^	11	9	< 0.001

Heart attack (%)	9 ^a, b^	3	1	< 0.001

Stroke (%)	4^d^	2	1	0.018

Heart failure (%)	7 ^a, b^	1	0	< 0.001

Arrhythmia (%)	12^a, d^	5	7	< 0.001

Osteoporosis (%)	14 ^a, b^	5	5	< 0.001

Diabetes (%)	10^b^	7	5	0.003

Inflammatory bowel disease (%)	5^e^	2	4	0.127

Peptic ulcer (%)	11^b^	7^d^	3	< 0.001

Reflux/heartburn (%)	27^d^	29^d^	19	0.031

Depression requiring tx (%)	17	15	14	0.506

**Physiology**				

FEV_1 _(% predicted)	48.3 ± 15.8 ^a, b^	108.6 ± 12.0^d^	114.8 ± 13.9	< 0.001

FEV_1_/FVC (%)	44.8 ± 11.6 ^a, b^	79.2 ± 5.2^d^	81.1 ± 5.2	< 0.001

FEV_1 _reversibility (%)	10.7 ± 13.7 ^a, b^	4.5 ± 5.8^d^	2.7 ± 4.5	< 0.001

Distance walked (metres)	369 ± 122			

BODE index	3.2 ± 2.1			

**Imaging**				

Emphysema (%)	17.6 ± 12.2 ^a, b^	2.4 ± 3.1^b^	4.1 ± 4.2	< 0.001

^a^p < 0.01 vs. smoking controls; ^b^< 0.01 vs. non-smoking controls; ^c ^In the year prior to study ^d^p < 0.05 vs. non-smoking controls. entry. ^e^p < 0.05 vs. smoking controls. **Abbreviations: **tx: treatment.

### Heterogeneity of COPD by severity of airflow limitation (GOLD)

**Figure 3:**
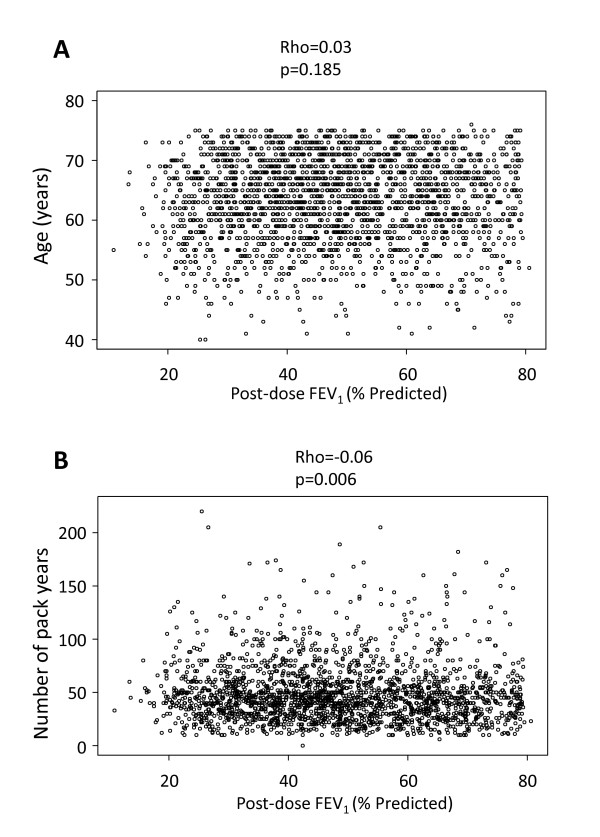
**Relationship between age (panel A) and cumulative smoking exposure (panel B) at entry into the study and degree of airflow limitation in patients with COPD**. For further explanations see text.

**Figure 4:**
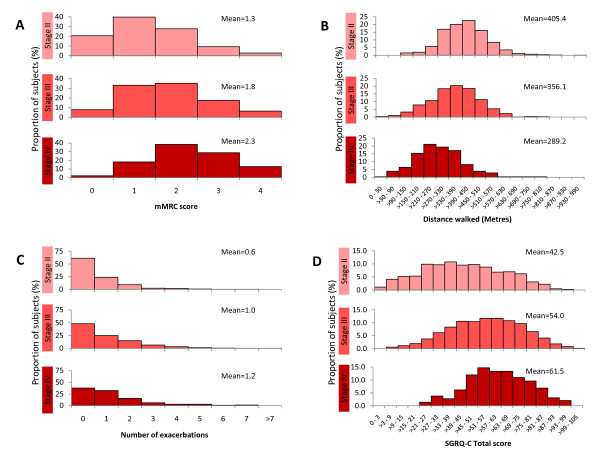
**Frequency distribution of the breathlessness as assessed by the mMRC questionnaire (panel A), exercise capacity as assessed by the 6MWD (panel B), reported exacerbations in the year before inclusion in the study (panel C), and health status assessed by SGRQ-C (panel D) according to severity of disease**. For further explanations see text.

**Figure 5:**
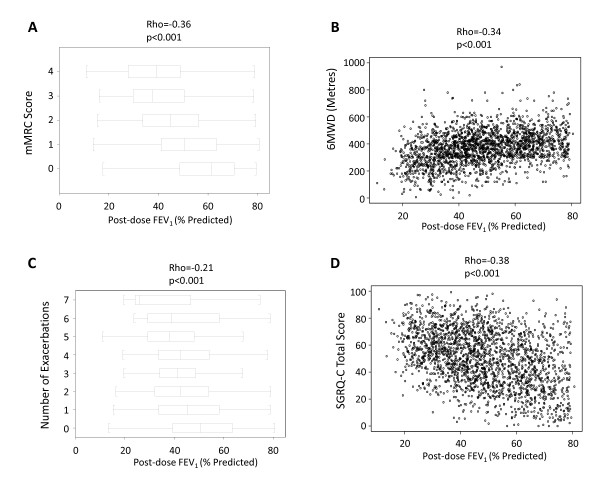
**Relationship between the severity of airflow limitation and breathlessness as assessed by the mMRC questionnaire (panel A), exercise capacity as assessed by the 6MWD (panel B), reported exacerbations in the year before inclusion in the study (panel C), and health status as assessed by SGRQ-C (panel D)**. For further explanations see text.

Age and pack-years of smoking were similar in the different GOLD stages (Table 2) and neither was related to the severity of airflow limitation (Figure 3). Symptoms (mMRC and SGRQ-C) and reported exacerbations during the previous year increased with disease severity, whereas the proportion of current smokers, BMI, FFMI, and the 6MWD decreased (Table 2). The frequency distribution of these variables within each GOLD category was wide and unimodal, so no discrete subgroups could be identified except for the fact that, within each GOLD stage, a substantial proportion of patients did not complain of symptoms, report exacerbations and/or exhibit exercise limitation, even with severe disease (Figure 4). In fact, while airflow limitation was significantly related to breathlessness, health status, 6MWD and number of exacerbations, there was considerable overlap between GOLD stages (Figure 5). FEV_1 _reversibility decreased in more severe disease. By contrast, co-morbidities appeared to be independent of the degree of airflow limitation (Table 2). The extent of emphysema (and the prevalence of bronchiectasis) increased in proportion to the GOLD stage (Table 2).

**Table 2:** Main anthropometric, clinical, functional, and radiological variables in patients with COPD, stratified according to disease severity (GOLD) and gender (mean ± SD, or proportion).

	GOLD II			GOLD III			GOLD IV			Comparing	
	**Females** **(n = 380)**	**Males** **(n = 574)**	**p value**	**Females** **(n = 293)**	**Males** **(n = 618)**	**p value**	**Females** **(n = 77)**	**Males** **(n = 219)**	**p value**	**GOLD stage within females**	**GOLD stage within males**

**Clinical Data**											

Age (years)	63.0 ± 7.1	63.8 ± 7.3	0.043	62.6 ± 6.8	64.2 ± 7.0	< 0.001	60.7 ± 6.8	63.0 ± 7.0	0.012	0.034	0.075

Pack-years	41.1 ± 21.6	52.7 ± 31.4	< 0.001	42.6 ± 21.2	52.2 ± 27.0	< 0.001	41.1 ± 21.8	52.1 ± 28.4	< 0.001	0.547	0.640

Current smokers (%)	40	36	0.300	37	38	0.695	27	28	0.922	0.114	0.027

BMI (kg/m^2^)	27.2 ± 6.4	27.5 ± 5.2	0.066	25.6 ± 6.0	26.4 ± 5.2	0.008	23.4 ± 6.4	25.5 ± 5.3	0.001	< 0.001	< 0.001

FFMI (kg/m^2^)	16.2 ± 3.0	18.4 ± 2.6	< 0.001	15.4 ± 2.3	17.8 ± 2.6	< 0.001	14.8 ± 2.5	17.0 ± 2.4	< 0.001	< 0.001	< 0.001

mMRC score	1.4 ± 1.0	1.3 ± 1.0	0.645	1.9 ± 1.0	1.8 ± 1.0	0.050	2.3 ± 1.0	2.3 ± 1.0	0.975	< 0.001	< 0.001

SGRQ-C (total)	43.8 ± 20.2	41.6 ± 20.9	0.193	55.4 ± 18.0	53.4 ± 18.5	0.215	61.3 ± 15.6	61.8 ± 16.1	0.885	< 0.001	< 0.001

Number of exacerbations^a^	0.8 ± 1.2	0.5 ± 0.9	< 0.001	1.2 ± 1.4	0.9 ± 1.3	0.005	1.5 ± 1.6	1.1 ± 1.4	0.044	< 0.001	< 0.001

Heart trouble (%)	19	30	< 0.001	17	30	< 0.001	22	27	0.343	0.632	0.687

Heart attack (%)	5	13	< 0.001	6	10	0.033	1	10	0.011	0.280	0.275

Stroke (%)	5	4	0.544	3	3	0.805	4	3	0.645	0.557	0.467

Heart failure (%)	4	9	0.002	3	8	0.003	11	9	0.597	0.007	0.884

Arrhythmia (%)	10	14	0.068	8	15	0.010	12	10	0.684	0.604	0.315

Osteoporosis (%)	28	5	< 0.001	32	7	< 0.001	29	7	< 0.001	0.601	0.415

Diabetes (%)	9	13	0.079	5	10	0.010	7	13	0.154	0.138	0.341

Inflammatory bowel disease (%)	9	4	0.003	6	3	0.016	12	4	0.019	0.156	0.308

Peptic ulcer (%)	10	12	0.283	10	11	0.728	11	7	0.239	0.959	0.082

Reflux/heartburn (%)	36	29	0.022	30	20	0.002	27	19	0.138	0.163	0.001

Depression requiring Tx (%)	23	11	< 0.001	32	10	< 0.001	26	12	0.004	0.036	0.846

**Physiology**											

FEV_1 _(% predicted)	63.6 ± 8.2	62.8 ± 8.5	0.119	41.0 ± 5.8	40.0 ± 5.8	0.017	25.4 ± 3.2	24.5 ± 3.8	0.156	< 0.001	< 0.001

FEV_1_/FVC (%)	53.2 ± 8.8	52.5 ± 8.8	0.251	41.4 ± 8.9	40.0 ± 8.9	0.021	34.5 ± 8.0	31.2 ± 7.2	0.002	< 0.001	< 0.001

FEV_1 _reversibility (%)	10.6 ± 12.1	11.7 ± 13.0	0.056	10.3 ± 14.8	11.4 ± 14.5	0.316	5.5 ± 13.2	8.9 ± 14.0	0.037	0.002	0.007

6MWD (metres)	391 ± 113	415 ± 110	0.003	333 ± 119	366 ± 116	< 0.001	265 ± 118	297 ± 119	0.069	< 0.001	< 0.001

BODE Index	1.7 ± 1.4	1.6 ± 1.3	0.715	4.2 ± 1.6	3.8 ± 1.6	< 0.001	6.0 ± 1.6	5.6 ± 1.6	0.079	< 0.001	< 0.001

**Imaging**											

Emphysema (%)	11.2 ± 9.5	12.7 ± 9.5	0.002	20.1 ± 11.7	20.0 ± 11.5	0.876	27.1 ± 13.7	28.6 ± 12.1	0.435	< 0.001	< 0.001

Bronchiectasis (%)	< 1	2	0.057	3	6	0.044	9	7	0.468	< 0.001	0.003

^a ^In the year prior to study entry.

**Abbreviations: **tx: treatment.

### Heterogeneity of COPD by the BODE index

As shown in table 3, when results were stratified according to the BODE index we found very similar results to those observed when disease severity was graded according to FEV_1 _(Table 2). Of note, age and pack-years of smoking were again similar in the different BODE scores. Symptoms (mMRC and SGRQ-C), airflow limitation, number of reported exacerbations and the extent of emphysema increased in proportion to BODE scores. By contrast, BMI, FFMI, and exercise tolerance decreased in proportion to BODE (Table 3).

**Table 3:** Main anthropometric, clinical, functional, and radiological variables in patients with COPD, stratified according to the BODE index and gender (mean ± SD or proportion).

	BODE 0 1 2			BODE 3 4			BODE 5 6			BODE 7 8 9 10			Differences within females	Differences within males
	**Females**	**Males**	**p value**	**Females**	**Males**	**p value**	**Females**	**Males**	**p value**	**Females**	**Males**	**p value**	**Females**	**Males**

BODE Index	1.1 ± 0.8	1.3 ± 0.8	0.014	3.5 ± 0.5	3.4 ± 0.5	0.088	5.4 ± 0.5	5.4 ± 0.5	0.874	7.5 ± 0.8	7.5 ± 0.7	0.658	< 0.001	< 0.001

	**Clinical data**													

Age (years)	62.3 ± 7.0	63.5 ± 7.3	0.008	62.8 ± 6.8	63.6 ± 7.2	0.132	62.3 ± 7.0	64.9 ± 6.6	0.001	63.9 ± 7.1	63.9 ± 7.0	0.949	0.362	0.090

Pack years (n)	39.7 ± 20.5	51.0 ± 29.2	< 0.001	43.3 ± 22.2	50.6 ± 27.7	0.001	43.6 ± 22.9	55.7 ± 28.0	< 0.001	41.9 ± 17.8	57.8 ± 31.8	< 0.001	0.186	0.002

BMI (Kg/m^2^)	26.4 ± 5.0	27.3 ± 4.5	0.001	26.1 ± 6.1	26.6 ± 5.5	0.162	26.4 ± 8.1	26.3 ± 6.1	0.596	23.8 ± 7.0	25.9 ± 6.0	0.017	0.006	< 0.001

FFMI (Kg/m^2^)	15.9 ± 2.7	18.4 ± 2.3	< 0.001	15.5 ± 2.3	17.9 ± 2.7	< 0.001	15.8 ± 3.0	17.6 ± 2.9	< 0.001	14.9 ± 3.0	17.1 ± 2.4	< 0.001	0.001	< 0.001

MMRC score	0.9 ± 0.7	0.9 ± 0.7	0.893	1.8 ± 0.7	1.8 ± 0.8	0.443	2.6 ± 0.9	2.5 ± 0.8	0.619	3.1 ± 0.8	3.3 ± 0.6	0.051	< 0.001	< 0.001

SGRQ total score	38.0 ± 17.1	38.0 ± 18.7	0.961	54.1 ± 15.5	53.0 ± 16.1	0.541	63.1 ± 16.1	62.6 ± 14.6	0.809	70.2 ± 12.9	72.5 ± 13.2	0.352	< 0.001	< 0.001

Exacerbations ^a^(n)	0.6 ± 1.0	0.5 ± 0.9	0.006	1.1 ± 1.4	0.9 ± 1.3	0.152	1.5 ± 1.4	1.2 ± 1.5	0.006	1.6 ± 1.7	1.1 ± 1.2	0.276	< 0.001	< 0.001

	**Physiology**													

Post-BD FEV1 (% ref).	62.1 ± 9.8	58.8 ± 11.7	< 0.001	46.6 ± 12.2	42.8 ± 12.1	0.001	37.9 ± 10.8	34.4 ± 9.4	0.003	32.6 ± 8.8	29.1 ± 8.5	0.022	< 0.001	< 0.001

Post-BD FEV/FVC (%)	51.9 ± 9.1	49.7 ± 10.0	0.003	44.5 ± 10.4	41.9 ± 11.1	0.004	40.0 ± 10.6	36.9 ± 9.9	0.009	39.7 ± 9.9	35.0 ± 8.6	0.003	< 0.001	< 0.001

FEV1 reversibility (%)	10.5 ± 12.5	11.3 ± 13.6	0.319	9.7 ± 14.8	12.2 ± 15.2	0.020	10.5 ± 14.4	10.6 ± 12.3	0.687	7.8 ± 12.8	8.3 ± 14.0	0.787	0.300	0.072

6MWD (metres)	432 ± 93	447 ± 94	0.013	349 ± 91	379 ± 95	< 0.001	269 ± 88	289 ± 84	0.015	164 ± 74	185 ± 67	0.084	< 0.001	< 0.001

	**Imaging**													

LAA	11.3 ± 9.9	13.8 ± 9.7	< 0.001	17.7 ± 11.1	19.0 ± 11.5	0.244	22.6 ± 13.2	23.6 ± 13.1	0.616	25.5 ± 13.6	28.9 ± 12.6	0.171	< 0.001	< 0.001

^a^In the year prior to study entry.

### Heterogeneity of COPD by gender

Within each GOLD category, age was similar between males and females, but the latter had less smoking exposure, lower BMI and FFMI, and reported more exacerbations than males (Table 2). Cardiovascular co-morbidity and diabetes appeared less prevalent in females, whereas osteoporosis, inflammatory bowel disease, reflux and depression requiring treatment were more reported more often by females. There were no obvious differences in the prevalence and/or severity of emphysema by gender within each GOLD stage.

When gender differences were analysed by BODE scores we found similar results. Age was similar between males and females but that the latter had less smoking exposure, lower BMI, FFMI and reported more exacerbations than males (Table 3). Reported symptoms and health status was similar between genders. Interestingly, spirometric indices at each BODE score were significantly higher in females than males, but exercise tolerance was lower (Table 3). There were no obvious gender differences by BODE in any of the radiological variables analysed.

### Heterogeneity of COPD by presence of chronic bronchitis

Table 4 presents the main clinical, functional and imaging variables by GOLD stages according to the presence of chronic bronchitis, which was defined as per the ATS questionnaire ("phlegm on most days for 3 or more consecutive months during the year and trouble with phlegm for 2 or more years"). We observed that for each GOLD stage there was a significant preponderance of current-smoker males among those with chronic bronchitis, and that these patients had a poorer health status than those without it (Table 4). No other clear and consistent signal, including lung function and imaging variables, could be identified. Interestingly, the frequency of reported exacerbations in the year before recruitment was not different, at each GOLD stage, between patients with and without chronic bronchitis (Table 4).

**Table 4:** Main anthropometric, clinical, functional, and radiological variables in patients with COPD, stratified according to disease severity (GOLD) and the presence or absence of chronic bronchitis (mean ± SD or proportion).

	GOLD II			GOLD III			GOLD IV			Comparing	
	**Yes**	**No**	**p-value**	**Yes**	**No**	**p-value**	**Yes**	**No**	**p-value**	**GOLD Stage within Yes**	**GOLD Stage within No**

**Clinical data**											

N	294	660		337	574		118	178			

Age (years)	62.6 (7.5)	63.8 (7.0)	0.018	63.2 (7.4)	64.0 (6.7)	0.183	62.0 (7.6)	62.7 (6.6)	0.698	0.335	0.043

Male (%)	193 (66%)	381 (58%)	0.021	258 (77%)	360 (63%)	< 0.001	96 (81%)	123 (69%)	0.019	< 0.001	0.014

Pack-years	49.0 (29.3)	47.7 (28.1)	0.423	50.3 (27.3)	48.5 (24.6)	0.450	50.1 (28.9)	48.7 (26.1)	0.870	0.578	0.172

Current smokers (%)	153 (52%)	207 (31%)	< 0.001	154 (46%)	187 (33%)	< 0.001	44 (37%)	38 (21%)	0.003	0.021	0.015

BMI (kg/m^2^)	26.7 (5.4)	27.7 (5.8)	0.007	26.0 (5.5)	26.3 (5.6)	0.415	24.3 (5.4)	25.4 (5.9)	0.140	< 0.001	< 0.001

FFMI (kg/m^2^)	17.4 (2.7)	17.6 (3.0)	0.339	17.0 (2.5)	17.0 (2.9)	0.387	16.4 (2.6)	16.4 (2.6)	0.986	0.008	< 0.001

mMRC Score	1.5 (1.0)	1.3 (1.0)	0.002	1.8 (1.0)	1.8 (1.1)	0.861	2.4 (1.0)	2.3 (1.0)	0.739	< 0.001	< 0.001

SGRQ-C total score	50.3 (18.8)	38.9 (20.5)	< 0.001	58.8 (17.6)	51.2 (18.2)	< 0.001	65.0 (16.5)	59.4 (15.2)	0.010	< 0.001	< 0.001

Number of exacerbations^a^	0.7 (1.1)	0.6 (1.0)	0.132	1.0 (1.2)	1.0 (1.4)	0.253	1.2 (1.6)	1.2 (1.3)	0.436	< 0.001	< 0.001

**Physiology**											

FEV1 % Predicted	62.3 (8.8)	63.5 (8.2)	0.030	40.3 (5.7)	40.3 (5.9)	0.957	25.1 (3.4)	24.5 (3.8)	0.322	< 0.001	< 0.001

FEV1/FVC (%)	52.3 (9.0)	53.0 (8.7)	0.358	41.0 (9.4)	40.1 (8.6)	0.199	32.2 (8.6)	31.9 (6.8)	0.985	< 0.001	< 0.001

FEV1 reversibility (%)	10.8 (12.4)	11.4 (12.8)	0.222	11.2 (15.1)	10.9 (14.3)	0.826	8.2 (15.3)	7.8 (12.9)	0.866	0.070	0.001

6MWD (metres)	397 (118)	409 (109)	0.059	352 (113)	358 (121)	0.289	306 (125)	278 (115)	0.106	< 0.001	< 0.001

BODE Index	1.8 (1.4)	1.5 (1.3)	< 0.001	4.0 (1.6)	3.9 (1.7)	0.430	5.7 (1.7)	5.8 (1.5)	0.730	< 0.001	< 0.001

**Imaging**											

Emphysema (%)	12.3 (10.3)	12.0 (9.2)	0.854	19.1 (11.7)	20.5 (11.5)	0.082	28.3 (13.4)	28.1 (12.1)	0.822	< 0.001	< 0.001

Bronchiectasis (%)	3 (1%)	10 (2%)	0.644	22 (8%)	19 (4%)	0.006	7 (7%)	13 (8%)	0.797	0.001	< 0.001

^a^In the year prior to study entry.

## Discussion

The results of this study confirm that COPD is a highly heterogeneous disease [2,3] and provide a number of observations that help to better delineate the complexity of the disease. Of particular clinical relevance is the observation that, within each GOLD stage (or BODE score) of disease severity, symptoms, exercise tolerance, the number of reported exacerbations and the prevalence of co-morbidities varied widely between patients, and that even in patients with severe airflow obstruction there were a substantial proportion of patients who did not complain of symptoms, report exacerbations or show impaired exercise tolerance. These observations highlight the fact that FEV_1 _does not capture the complexity of the disease and that clinical management of patients with COPD needs to consider such complexity rather than just spirometry alone. Other observations of interest are discussed below.

COPD is characterised by an accelerated rate of decline of FEV_1 _with age [1,12]. According to this model, one might expect patients with severe COPD to be older. This was not the case in ECLIPSE. In fact, we did not find any relationship between age and FEV_1_. Several explanations can be conceived for this, apparently odd, observation. On the one hand, it should be explicitly acknowledged that ECLIPSE is not a population-based study. Hence, this observation can be due to sampling bias, as compared to the epidemiological studies where most of the conflicting data comes from [12]. Thus, factors relating to subject recruitment into ECLIPSE may have resulted in similarly aged subjects regardless of severity being enrolled. On the other hand, the lack of relationship between age and severity of airflow limitation may also indicate that the ECLIPSE subjects had previously had a wide range of lung function decline, a possibility that would be perfectly in line with the accepted pathophysiological models of COPD [1,12] because it would suggest that, similarly to what has been described in idiopathic pulmonary fibrosis [13] and has been suggested in COPD [14,15], there are likely to be rapid and slow decliners among the population of COPD patients at large. This hypothesis will be tested directly in the three-year longitudinal portion of ECLIPSE.

Tobacco smoking is the main risk factor for COPD [1] but it is well established that not all smokers develop the disease as indicated by the identification of susceptible and non-susceptible smokers [12]. We observed that, even among susceptible smokers (i.e. those smokers who have already developed COPD), the relationship between smoking exposure, as gauged by self-reported cigarette use, and airflow limitation is poor, albeit statistically significant (Figure 3, panel B). This suggests that 'susceptibility' is not a yes-no phenomenon. In fact, a range of 'susceptibility levels' was already suggested by Fletcher and Peto in 1977 [12], and has been more recently confirmed in the Framingham offspring cohort [16], potentially reflecting genetic differences or interactions with other risk factors, such as nutrition or infections. However, similarly to what we discussed above in relation to age, because ECLIPSE is not a population-based study, a potential sampling bias cannot be excluded. Likewise, smoking exposure was assessed by self-reported pack-years, which is known to be a very crude estimate of cumulative exposure to smoking.

Relief of symptoms and prevention of exacerbations are two of the main goals of COPD management [1]. To achieve them, therapy in COPD is guided broadly by the severity of airflow limitation [1]. We confirmed [17] that airflow limitation was poorly related to the degree of breathlessness, health status, 6MWD and number of exacerbations reported in the year before the study (Figure 4). Furthermore, as discussed above, within each category of airflow limitation, mMRC, SGRQ-C, 6MWD and the number of reported exacerbations varied widely. In fact, a substantial percentage of patients with severe airflow obstruction did not complain of symptoms, report exacerbations or show impaired exercise tolerance (Figure 4). These observations support the strategy suggested by the UK National Institute for Clinical Excellence (NICE) in that symptoms, exacerbations and co-morbidities must also be included in the assessment of the severity in any given patient, rather than just spirometry alone, because this is likely to offer a more appropriate way to direct therapy [18,19].

We found that the frequency of reported exacerbations increased in parallel with airflow limitation but, interestingly, exacerbations were not reported by a substantial proportion of patients, including those with severe disease (Figure 4, panel C). Because this observation was based on patient recall, it may be subject to both selection and recall bias. Observations in relation to exacerbations, therefore, need to be confirmed prospectively during follow-up in ECLIPSE. If confirmed, a deeper understanding of why some patients with COPD develop exacerbations, which may represent a phenotype of COPD, whereas others do not (which may represent another one) despite a similar degree of airflow limitation may emerge. We could not confirm previous observations [20] that exacerbations of COPD were more prevalent among patients with chronic bronchitis, at each GOLD stage (Table 4). Yet, these are self-reported exacerbations over the previous year, and this may be subject to recall bias, so this finding will have to be confirmed or refuted during prospective follow up of these patients.

In keeping with previous results [21], we found that BMI and FFMI decreased progressively with increasing airflow limitation, particularly in females (Table 2). Likewise, our results also confirm that co-morbidities occur more frequently in patients with COPD than in controls [22]. However, co-morbidities were largely independent of the degree of airflow limitation and occurred similarly in both moderate and severe disease. Because, ECLIPSE is not a population-based study, we cannot exclude some type of selection bias. However, the possibility that co-morbidities may occur early during the course of the disease raises important questions about their potential pathogenic mechanisms [22] and highlights the clinical importance of identifying (and treating) them if present early in the course of the disease. We also found that the extent of emphysema increased with GOLD stage, as did the proportion of COPD patients with bronchiectasis, although this proportion was small and lower than that reported previously [23].

We identified several, potentially relevant, gender differences. Interestingly, females had less smoking exposure for the same degree of airflow limitation (Table 2) or BODE score (Table 3), suggesting that women are more susceptible to tobacco smoke. This observation supports previous studies [24]. Also in keeping with previous reports [25], we found that women reported more exacerbations than males for the same GOLD stage (Table 2). Finally, females with COPD appeared particularly susceptible to osteoporosis, inflammatory bowel disease, reflux and depression requiring treatment relative to males, but less so with respect to cardiovascular co-morbidity and diabetes (Table 2).

The main strength of our study is the large sample size of patients (and controls) included, as well as their careful clinical and functional characterisation thus allowing the study of relationships between clinical, functional, and radiological variables. The size of ECLIPSE permits more accurate estimates of the variance observed in a number of key parameters used to assess COPD patients. While ECLIPSE is not a population-based sample, recruitment was very similar to that in other clinical trials and variability between centres was minor (Figure 2). Thus, the data generated in ECLIPSE will be helpful in estimating sample sizes in future clinical studies. Our study, however, has some limitations. First, COPD patients were older than controls and had a history of more intense smoking exposure than the group of smokers with normal lung function. This may limit some of the comparisons between patients and controls, but it is not relevant for those analyses that include COPD patients only. Second, many patients were recruited from populations receiving care at the participating hospitals. Thus, the population studied here may not be a true reflection of the COPD patients regularly seen in primary care.

## Conclusions

In summary, our results help to better delineate the heterogeneity and complexity of COPD by describing the relationships (or lack thereof) between a number of important clinical, functional, and radiological domains of the disease. Of potential particular relevance is our finding that the current GOLD classification of disease severity, based upon the degree of airflow limitation, is a poor predictor of other features of COPD. This observation is in keeping with recent observations by Burgel *et al *using principal component analysis and cluster analysis in a cohort of COPD subjects recruited in a French multicentre study [26]. The clinical utility of the subgroups identified in any cross-sectional analysis, however, needs to be validated longitudinally against clinically relevant outcomes [27]. The longitudinal analysis of the follow-up data of the patients included in the ECLIPSE study should hopefully allow these goals to be achieved and, with them, a better understanding of the complexity of the disease and potential clinical relevance of the identified phenotypes.

## Competing interests

AA has received reimbursements, fees, or funding from GlaxoSmithKline, Almirall, AstraZeneca, Boheringer-Ingelheim, Roche, Nycomed, Novartis and Procter & Gamble; PMAC has received consulting fees from AstraZeneca, GlaxoSmithKline, Nycomed and Pfizer, speaking fees from GlaxoSmithKline and Nycomed; and grant support from Boehringer-Ingelheim and GlaxoSmithKline; BC has received grants to the pulmonary division he works in to complete research studies. From GlaxoSmithKline, Boehringer-Ingelheim, Forrest Medical, Astra Zeneca and Aeris; has served on advisory boards for GlaxoSmithKline, Boehringer-Ingelheim, Almirall, Astra Zeneca, Aeris and Deep Breeze; has received speaker fees from GlaxoSmithKline, Boehringer-Ingelheim, Astra Zeneca, Almirall and Esteve; Does not have shares or interest in any company. Neither does the family; Has not received tobacco money nor has stocks in any tobacco related companies; HOC has received an honorarium for serving on the steering committee for the ECLIPSE project for GlaxoSmithKline. In addition HC was the co-investigator on two multi-centre studies sponsored by GlaxoSmithKline and has received travel expenses to attend meetings related to the project. HC has three contract service agreements with GlaxoSmithKline to quantify the CT scans in subjects with COPD and a service agreement with Spiration Inc to measure changes in lung volume in subjects with severe emphysema HC is the co-investigator (D Sin PI) on a Canadian Institutes of Health - Industry (Wyeth) partnership grant. HC has received a fee for speaking at a conference and related travel expenses from AstraZeneca (Australia); LDE is an employee of GlaxoSmithKline and hold stocks and stock options in GlaxoSmithKline; DAL has received grant funding, honoraria and travel expenses from GlaxoSmithKline and serves on the Respiratory CEDD Board of GlaxoSmithKline; WM has been reimbursed for travel by GlaxoSmithKline, Zambon, Astra Zeneca, Boehringer-Ingelheim, Pfizer and Micromet for attending conferences; has received honoraria from GlaxoSmithKline and AstraZeneca for participating as a speaker in scientific meetings; serves on advisory boards for GlaxoSmithKline, Pfizer, Almirall, Amgen, Bayer and Micromet; serves as a consultant for Pfizer and SMB Pharmaceuticals; BEM is an employee and shareholder of GlaxoSmithKline, the sponsor of ECLIPSE; SR has consulted or participated in advisory boards for: Able Associates, Adelphia Research, Almirall/Prescott, APT Pharma/Britnall, Aradigm, AstraZeneca, Boehringer-Ingelheim, Chiesi, CommonHealth, Consult Complete, COP Forum, DataMonitor, Decision Resources, Defined Health, Dey, Dunn Group, Eaton Associates, Equinox, Gerson, GlaxoSmithKline, Infomed, KOL Connection, M. Pankove, MedaCorp, MDRx Financial, Mpex, Novartis, Nycomed, Oriel Therapeutics, Otsuka, Pennside Partners, Pfizer (Varenicline), PharmaVentures, Pharmaxis, Price Waterhouse, Propagate, Pulmatrix, Reckner Associates, Recruiting Resources, Roche, Schlesinger Medical, Scimed, Sudler and Hennessey, TargeGen, Theravance, UBC, Uptake Medical, VantagePoint Management. SR has given lectures for: American Thoracic Society, Astra Zeneca, Boehringer-Ingelheim, California Allergy Society, Creative Educational Concept, France Foundation, Information TV, Network for Continuing Ed, Novartis, Pfizer, SOMA. SR has received industry-sponsored grants from: Astra Zeneca, Biomarck, Centocor, Mpex, Nabi, Novartis, Otsuka; EKS has received grant support and consulting fees from GlaxoSmithKline for studies of COPD genetics and honoraria and consulting fees from Astra Zeneca; RT-S is an employee and shareholder of GlaxoSmithKline, the sponsor of ECLIPSE; EW serves on an advisory board for Nycomed; has received lecture fees from GlaxoSmithKline, Astra Zeneca and Novartis; has received research grants from GlaxoSmithKline and Astra Zeneca; JCY is an employee and shareholder of GlaxoSmithKline, the sponsor of ECLIPSE; JV has received fees for advising and/or presenting from GlaxoSmithKline, Astra Zeneca, Pfizer, Boehringer-Ingelheim, Nycomed, Hofmann - la Roche, Talecris, Kamada and Sounds Biotech; has received research support from GlaxoSmithKline.

## Authors' contributions

The authors developed the design and concept of the study, approved the statistical plan, had full access to and interpreted the data, wrote the article, read and approved the final manuscript and were responsible for decisions with regard to publication.

AA was a study investigator, developed the study protocol, served on the scientific committee, interpreted study data, developed the first draft of the manuscript, contributed to and reviewed drafts of the manuscript, and approved the final version of the manuscript; PMAC developed the study protocol, served on the scientific committee, interpreted study data, contributed to and reviewed drafts of the manuscript, and approved the final version of the manuscript; BC was a study investigator, developed the study protocol, served on the scientific committee, interpreted study data, contributed to and reviewed drafts of the manuscript, and approved the final version of the manuscript; HOC developed the study protocol, served on the steering committee, interpreted study data, contributed to and reviewed drafts of the manuscript, and approved the final version of the manuscript; LDE developed the study protocol, served on the steering committee, performed statistical analysis and interpreted data, contributed to and reviewed drafts of the manuscript, and approved the final version of the manuscript; DAL developed the study protocol, served on the steering committee, interpreted study data, contributed to and reviewed drafts of the manuscript, and approved the final version of the manuscript. WM was a study investigator, developed the study protocol, served on the steering and scientific (Chair) committees, interpreted study data, contributed to and reviewed drafts of the manuscript, and approved the final version of the manuscript; BEM interpreted data, contributed to and reviewed drafts of the manuscript, and approved the final version of the manuscript; SR was a study investigator, developed the study protocol, served on the scientific committee, interpreted study data, contributed to and reviewed drafts of the manuscript, and approved the final version of the manuscript; EKS was a study investigator, developed the study protocol, served on the steering committee, interpreted study data, contributed to and reviewed drafts of the manuscript, and approved the final version of the manuscript; RTS developed the study protocol, served on the steering and scientific committees, interpreted study data, contributed to and reviewed drafts of the manuscript, and approved the final version of the manuscript; EW served on the scientific committee, developed the study protocol interpreted study data, contributed to and reviewed drafts of the manuscript, and approved the final version of the manuscript; JCY developed the study protocol, served on the steering and scientific committees, interpreted study data, contributed to and reviewed drafts of the manuscript, and approved the final version of the manuscript. JV was a study investigator, developed the study protocol, served on the steering committee, interpreted study data, contributed to and reviewed drafts of the manuscript, and approved the final version of the manuscript.

The study sponsor (GlaxoSmithKline) did not place any restrictions with regard to statements made in the final version of the article.

## Appendix

PRINCIPAL INVESTIGATORS AND CENTRES PARTICIPATING IN ECLIPSE (NCT00292552, SC0104960)

Bulgaria: Y Ivanov, Pleven; K Kostov, Sofia. Canada: J Bourbeau, Montreal; M Fitzgerald, Vancouver; P Hernández, Halifax; K Killian, Hamilton; R Levy, Vancouver; F Maltais, Montreal; D O'Donnell, Kingston. Czech Republic: J Krepelka, Praha. Denmark: J Vestbo, Hvidovre. The Netherlands: E Wouters, Horn. New Zealand: D Quinn, Wellington. Norway: P Bakke, Bergen, Slovenia: M Kosnik, Golnik. Spain: A Agusti, Jaume Sauleda, Palma de Mallorca. Ukraine: Y Feschenko, Kiev; V Gavrisyuk, Kiev; L Yashina, W MacNee, Edinburgh; D Singh, Manchester; J Wedzicha, London. USA: A Anzueto, San Antonio, TX; S Braman, Providence. RI; R Casaburi, Torrance CA; B Celli, Boston, MA; G Giessel, Richmond, VA; M Gotfried, Phoenix, AZ; G Greenwald, Rancho Mirage, CA; N Hanania, Houston, TX; D Mahler, Lebanon, NH; B Make, Denver, CO; S Rennard, Omaha, NE; C Rochester, New Haven, CT; P Scanlon, Rochester, MN; D Schuller, Omaha, NE; F Sciurba, Pittsburg, PA; A Sharafkhaneh, Houston, TX; T Siler, St Charles, MO; E Silverman, Boston, MA; A Wanner, Miami, FL; R Wise, Baltimore, MD; R ZuWallack, Hartford, CT.

**Steering Committee: **H Coxson (Canada), C Crim (GlaxoSmithKline, USA), L Edwards (GlaxoSmithKline, USA), D Lomas (UK), W MacNee (UK), E Silverman (USA), R Tal-Singer (Co-chair, GlaxoSmithKline, USA), J Vestbo (Co-chair, Denmark), J Yates (GlaxoSmithKline, USA).

**Scientific Committee: **A Agusti (Spain), P Calverley (UK), B Celli (USA), C Crim (GlaxoSmithKline, USA), B Miller(GlaxoSmithKline, US), W MacNee (Chair, UK), S Rennard (USA), R Tal-Singer (GlaxoSmithKline, USA), E Wouters (The Netherlands), J Yates (GlaxoSmithKline, USA).
